# Erdheim-Chester Disease With BRAF V600E Mutation and Central Diabetes Insipidus Successfully Treated With Glucocorticoid

**DOI:** 10.1210/jcemcr/luad014

**Published:** 2023-03-17

**Authors:** Toshinori Imaizumi, Hisashi Daido, Takehiro Kato, Daisuke Yabe

**Affiliations:** Department of Diabetes, Endocrinology and Metabolism, Gifu University Graduate School of Medicine, Gifu 501-1194, Japan; Department of Rheumatology and Clinical Immunology, Gifu University Graduate School of Medicine, Gifu 501-1194, Japan; Department of Diabetes and Endocrinology, Gifu Prefectural General Medical Center, Gifu 500-8717, Japan; Department of Diabetes and Endocrinology, Gifu Prefectural General Medical Center, Gifu 500-8717, Japan; Department of Diabetes, Endocrinology and Metabolism, Gifu University Graduate School of Medicine, Gifu 501-1194, Japan; Department of Rheumatology and Clinical Immunology, Gifu University Graduate School of Medicine, Gifu 501-1194, Japan; Department of Diabetes, Endocrinology and Metabolism, Gifu University Graduate School of Medicine, Gifu 501-1194, Japan; Department of Rheumatology and Clinical Immunology, Gifu University Graduate School of Medicine, Gifu 501-1194, Japan; Center for One Medicine Innovative Translational Research, Gifu University Institute for Advanced Studies, Gifu 501-1194, Japan; Preemptive Food Research Center, Gifu University Institute for Advanced Studies, Gifu 501-1194, Japan; Center for Healthcare Information Technology, Tokai National Higher Education and Research System, Nagoya 464-8601, Japan

**Keywords:** Erdheim-Chester disease, central diabetes insipidus, retroperitoneal lesion, kidney dysfunction

## Abstract

Erdheim-Chester disease (ECD) is a rare non-Langerhans cell histiocytosis characterized by xanthoma/xanthogranuloma infiltration in various organs and a broad spectrum of clinical presentations, including bone lesions, central diabetes insipidus and renal failure. BRAF V600E mutation is seen in almost half of the cases of ECD; the BRAF inhibitor vemurafenib is recommended treatment in the United States and the European Union. However, the indication for vemurafenib in Japan is limited to unresectable malignant melanoma with BRAF mutation. Although glucocorticoids, interferon, chemotherapy, and radiation therapy are treatment options, no standard therapy for ECD has yet been established in Japan. We describe here a patient with central diabetes insipidus and retroperitoneal lesions who was successfully treated with prednisolone. Glucocorticoid therapy is therefore a plausible alternative for ECD with BRAF V600E mutation when the BRAF inhibitor vemurafenib cannot be used.

## Introduction

Erdheim-Chester disease (ECD) is a rare non-Langerhans cell histiocytosis of unknown etiology first described by William Chester in 1930 [1]. ECD is characterized by xanthoma/xanthogranuloma infiltration in various organs and a broad spectrum of clinical presentations, including bone lesions, central diabetes insipidus (CDI) and renal failure [2]. CDI is a relatively common endocrine disorder in ECD, occurring in 25% to 50% of patients, and is characterized by pituitary stalk enlargement on magnetic resonance imaging (MRI) [2]. “Hairy kidney,” hydronephrosis, and ureteral stricture associated with perinephric tissue involvement are also common, and retroperitoneal lesions are seen in up to 50% to 60% of patients [2]. When hydronephrosis is present with renal dysfunction, ureteral stenting or nephrostomy may be necessary [2]; biopsy of the lesions shows foamy histiocytes positive for CD68 and multinucleated giant cells negative for CD1a and S100 [2]. BRAF V600E mutation is an activating mutation of proto-oncogene BRAF that activates the mitogen-activated protein kinase (MAPK) pathway, which recently prompted recognition of ECD as a neoplastic disorder [2]. The mutation, which is seen in almost half of the cases of ECD, is reported to be associated with kidney, adrenal, and abdominal aortic lesions [3]. For symptomatic ECD with BRAF V600E mutation, the BRAF inhibitor vemurafenib has been approved by the Food and Drug Administration in the United States and by the European Medicines Agency in Europe but not by the Pharmaceuticals and Medical Devices Agency in Japan, where ECD with BRAF V600E mutation may be treated by alternatives including glucocorticoid [2, 4]. We describe here a patient with CDI and ECD who was successfully treated with prednisolone.

## Case Presentation

A 21-year-old man noticed thirst, polydipsia (6-7 L/day), and polyuria. At the age of 26, he became aware of lower abdominal pain and consulted his family doctor. Ultrasonography revealed bladder dilatation and bilateral hydronephrosis. He was then referred to our institution.

## Diagnostic Assessment

**Figure 1.**
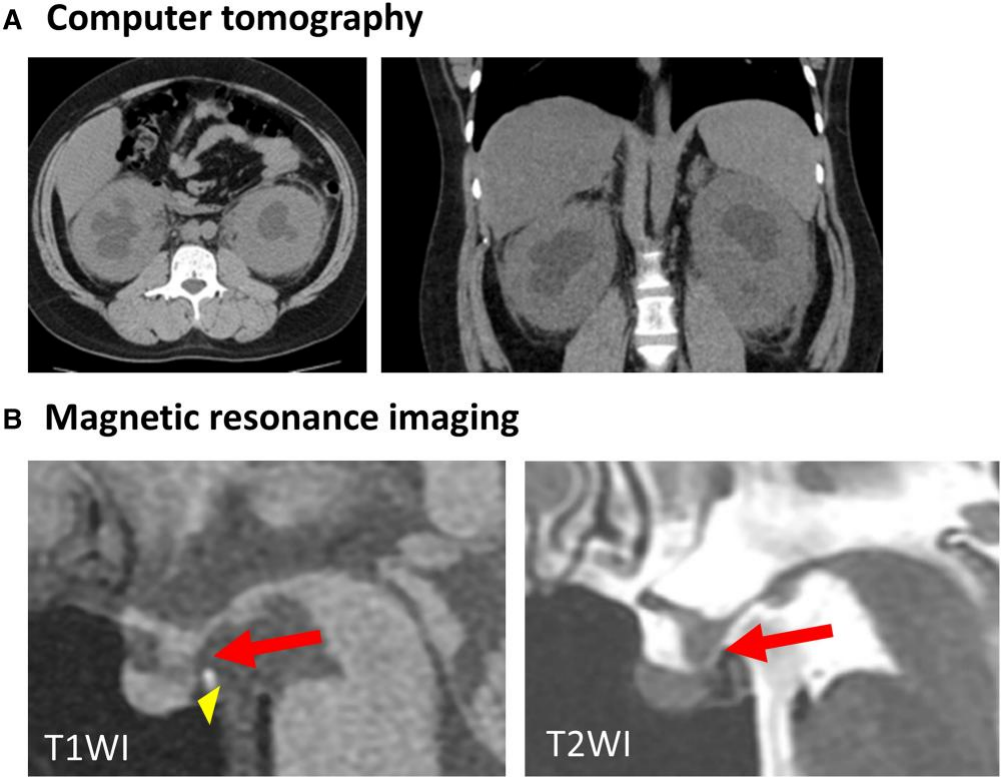
A, Abdominal plain computed tomography before treatment. “Hairy kidney” can be seen in bilateral kidneys. B, Pituitary magnetic resonance imaging before treatment. Pituitary stalk thickness (red arrow) and loss of high-intensity area on T1WI (yellow arrowhead) are apparent.

**Figure 2.**
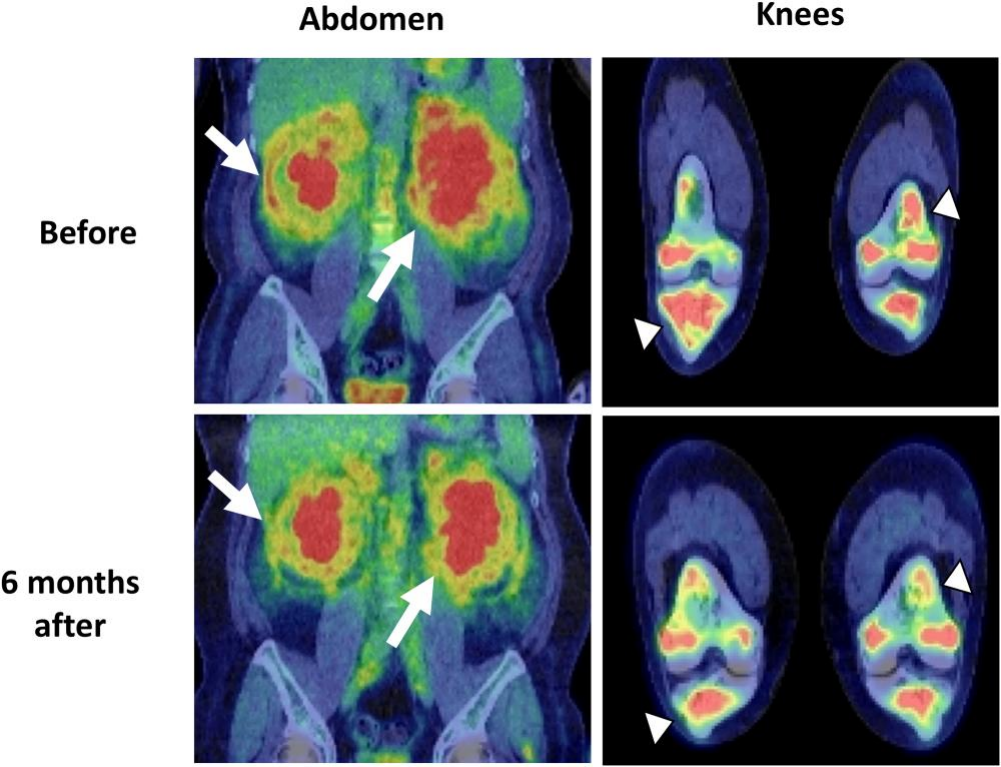
^18^F-fluorodeoxyglucose (FDG)-positron emission tomography (PET) before and 6 months after treatment. Improvement of SUV max in the abdominal lesions (from 4.8 to 3.8) (arrow) and knees (from 9.68 to 7.20) (arrowhead) can be seen.

**Figure 3.**
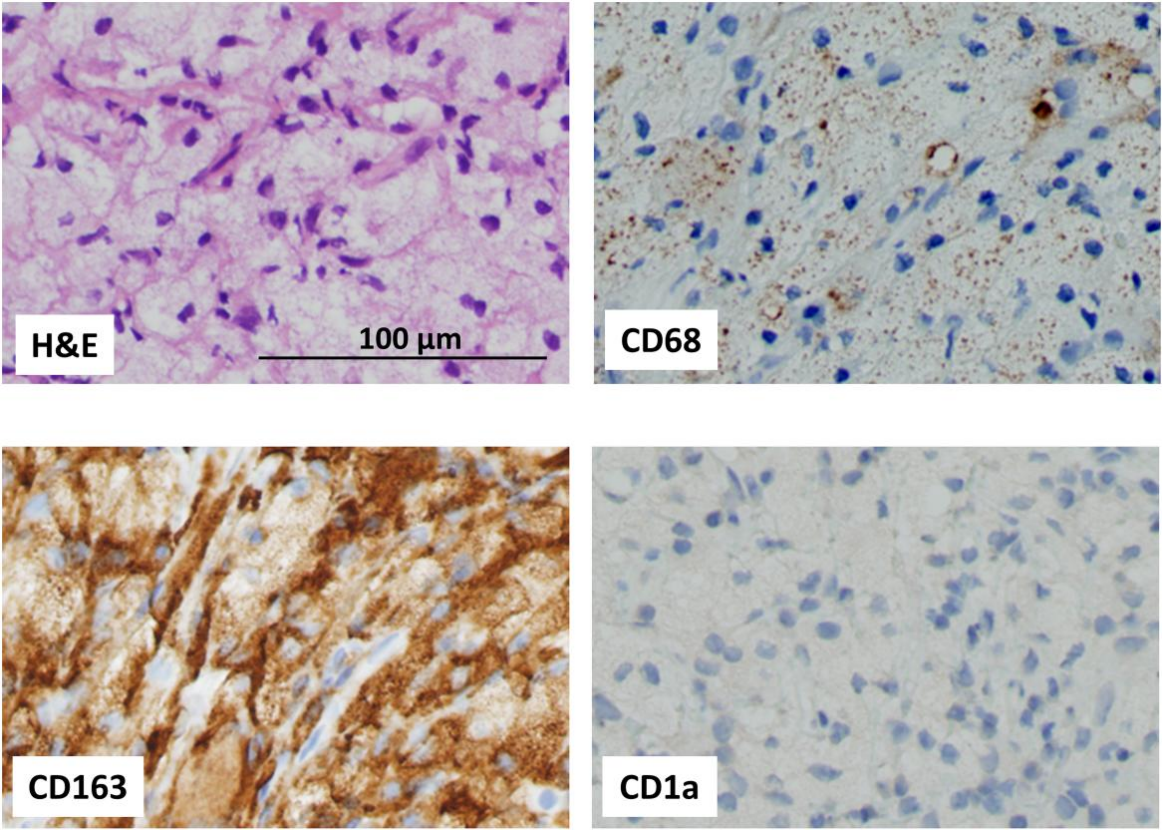
Pathological findings of axillary adipose tissue show histiocytes with abundant foamy and xanthomatous cytoplasm. Immunohistochemical staining shows positivity for CD68 and CD163 and negativity for CD1a. BRAF V600E mutation was confirmed by real-time PCR in biopsy sample. Abbreviations: H&E, hematoxylin and eosin stain; CD, cluster of differentiation.

The patient’s serum creatinine of 3.26 mg/dL (288.2 µmol/L), blood urea nitrogen of 29 mg/dL (10.35 mmol/L), polyuria of 4 to 10 L/day, urinary osmolality of 151 mOsm/kg, with no increase in plasma antidiuretic hormone (ADH) in hypertonic saline load test led to diagnosis of kidney failure and CDI (Table 1). While hyperprolactinemia was observed, other anterior pituitary functions were preserved. On transthoracic echocardiography, the ejection fraction was decreased (54%) while right atrium pseudotumor was not observed. Swelling of the stalk in pituitary MRI, “hairy kidney” in abdominal computed tomography (CT) and increased ^18^F-fluorodeoxyglucose (FDG) uptake in knees, perirenal lesions, skull, axillary adipose tissue, and sacral vertebral spinal canal in FDG-positron emission tomography (PET) suggested ECD (Figs. 1 and 2). Biopsy of the perirenal lesions and axillary adipose tissue showed histiocytes with abundant foamy and xanthomatous cytoplasm (Fig. 3). Immunohistochemical staining in axillary adipose tissue showed positivity for CD68 and CD163 and negativity for CD1a (Fig. 3). BRAF V600E mutation was detected by real-time polymerase chain reaction in biopsy sample. These findings prompted diagnosis of ECD.

**Table 1.** Biochemistry, urinalysis and basal levels of various hormones

Biochemistry	Value	Reference range
Na	146 mmol/L	138–149
Osmolality	301 mOsm/kg	275–290
BUN	29 mg/dL (10.35 mmol/L)	8–20 (2.86–7.14)
Cre	3.26 mg/dL (288.2 µmol/L)	0.65–1.07 (57.5–94.6)
eGFR	21.7 mL/min/1.73 m^2^	
CRP	10.63 mg/dL (106 300 ug/L)	0.00–0.14 (0–1400)
**Urinalysis**		
Osmolality	151 mOsm/kg	50–1200
**Various hormones**		
ACTH	28.9 pg/mL (6.36 pmol/L)	7.2–63.3 (1.58–13.9)
Cortisol	14.8 µg/dL (408.5 nmol/L)	4.5–21.1 (124.2–582.4)
TSH	1.445 µIU/mL (1.445 mU/L)	0.541–4.261 (0.541–4.261)
Free T3	3.19 pg/mL (4.90 pmol/L)	2.39–4.06 (3.67–6.24)
Free T4	1.06 ng/dL (0.14 pmol/L)	0.76–1.65 (0.10–0.21)
PRL	56.9 ng/mL (56.9 µg/L)	3.6–12.8 (3.6–12.8)
GH	0.30 ng/mL (0.30 µg/L)	0.00–2.47 (0.00–2.47)
IGF-1	100 ng/mL (13.1 nmol/L)	119–329 (15.6–43.1)
LH	4.0 mIU/mL (4.0 U/L)	0.8–5.7 (0.8–5.7)
FSH	1.8 mIU/mL (1.8 U/L)	2.0–8.3 (2.0–8.3)
Testosterone	1.3 ng/mL (4.5 nmol/L)	1.4–9.2 (4.9–31.9)
ADH	0.7 pg/mL (0.6 pmol/L)	

Values in parentheses are Système International. Abbreviations: ACTH, adrenocorticotropic hormone; BUN, blood urea nitrogen; Cre, creatinine; CRP, C-reactive protein; eGFR, estimated glomerular filtration rate; FSH, follicle-stimulating hormone; GH, growth hormone; IGF-1, insulin-like growth factor-1; LH, luteinizing hormone; PRL, prolactin; T3, triiodothyronine; T4, thyroxine; TSH, thyroid-stimulating hormone.

## Treatment

**Figure 4.**
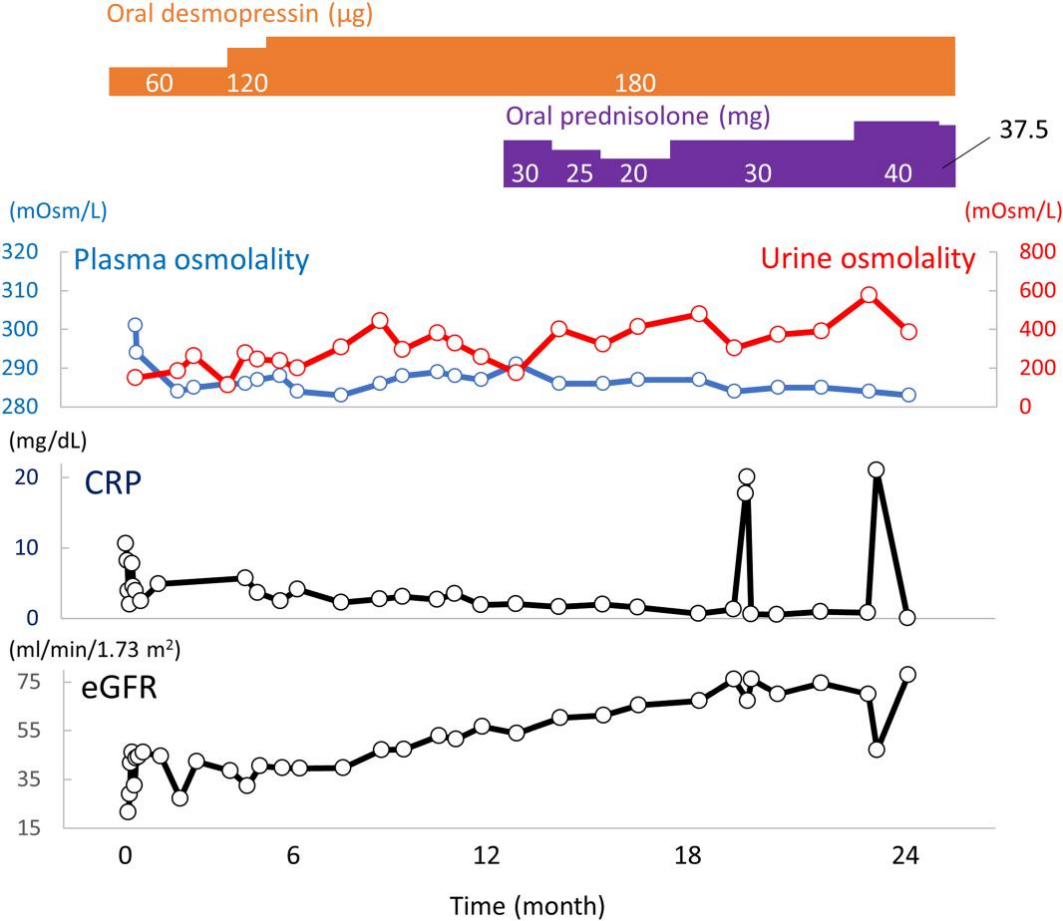
Time course of treatment. After treatment of diabetes insipidus with oral desmopressin was initiated, polyuria and diluted urine improved, and there was a trend toward improved renal function. After initiation of glucocorticoid, the trend to improved renal function continued, the desmopressin requirement remaining unchanged.

Although vemurafenib is first-line therapy for ECD with BRAF V600E mutation in the United States and Europe, it is not approved in Japan. Other therapies such as MEK inhibitors, mTOR inhibitors, interferon-alpha, and cytotoxic chemotherapies are also not approved in Japan. Our patient did not consent to the use of any of these drugs because of the cost burden. As glucocorticoid is an inexpensive drug for diseases requiring inflammation control, we initiated oral prednisolone 30 mg per day (0.3 mg/kg/day) to be adjusted according to subsequent clinical manifestations including abdominal pain and C-reactive protein level (Fig. 4). Oral desmopressin treatment was initiated earlier to treat the CDI.

## Outcome and Follow-up

Kidney dysfunction, polyuria, and bladder dilation as well as dilute urine gradually improved (Fig. 4). In addition, FDG-PET showed improvement of maximum standardized uptake value (SUV max) in the perirenal lesions (from 4.8 to 3.8) and knees (from 9.68 to 7.20) (Fig. 2) while pituitary stalk thickness and hydronephrosis remained unchanged. To date, the patient shows no significant disease progression suggesting fatal complications. As the patient had better appetite and showed weight gain, we tapered prednisolone 2.5 to 5.0 mg per month with the goal of achieving the lowest dose that suppresses clinical symptoms of ECD such as abdominal pain, inflammation, and kidney dysfunction. No other major adverse events have been observed so far.

## Discussion

ECD is a rare disease; only ∼800 cases have been reported in the literature globally [2]. A nationwide survey in Japan in 2018 found 75 ECD previous cases [4]. Bone is the most frequently involved organ and typically shows meta-diaphyseal osteosclerosis (95% of cases [2]), such as the knee lesions seen in our patient. Involvement of retroperitoneum is 50% to 60% and that of pituitary is 40% to 70% [2], both of which occurred in the present case. Kidney is involved in 65% of cases [2], renal failure being due to direct invasion of the renal sinus and parenchyma, distal ureteral obstruction with perirenal lesions, and hydronephrosis or kidney ischemic injury [2]. Some patients require nephrostomy or ureteral stenting for hydronephrosis [2]. Among the cases of pituitary involvement in ECD, CDI is the most common, and may precede diagnosis of ECD by several years or even decades [2]. Hyperprolactinemia is seen 15% to 30% of cases, which is possibly due to disruption of the hypothalamic dopaminergic pathway or kidney dysfunction in our patient [2]. Indicating worse morbidity and mortality, heart involvement (37%) and that of the central nervous system (38%) also occur [2]. Median survival from the initial onset of ECD is about 10 years, most cases being progressive and fatal within a few years [4].

In the recent consensus recommendation, BRAF inhibitor therapy is endorsed as first-line therapy for ECD patients with BRAF V600E mutation who also manifest cardiac/neurologic disease or end-organ dysfunction [2]. On the other hand, for patients with low-burden disease involving bones and retroperitoneum or without access to targeted therapies, interferon-alpha/peg-interferon-alpha, cladribine, or anakinra may be considered [2]. Glucocorticoid is not recommended as first-line therapy for ECD because analysis of its survival benefit is scant [2]. However, glucocorticoid might be expected to be effective against the inflammatory features of ECD because of its reported alleviation of symptoms related to tissue swelling, such as those in orbital disease, as well as its clinical efficacy in improving lesions of kidney and bone [2, 5]. Although the mechanism by which glucocorticoid might exert its efficacy is unclear, an activated MAPK pathway is linked to the senescence-associated secretory phenotype, which could lead to a Th1-oriented inflammatory response capable of worsening the lesions, suggesting inhibition of Th1 as a possible mechanism of beneficial effect [6, 7]. On the other hand, it should be noted that glucocorticoid may exhibit a variety of side effects, especially susceptibility to infection, which can be fatal in ECD [4]. In the present case, glucocorticoid administration was followed by improved renal function and FDG uptake in the retroperitoneal lesions. Kidney function assessment is recommended to be included in newly diagnosed ECD, and FDG-PET is considered the optimal modality for ECD response assessment; however, reports on kidney function and FDG-PET after glucocorticoid use are scarce [2]. Because there is no standard protocol for adjusting glucocorticoid dosage in ECD, we adjusted the dose of prednisolone based on the patient's subsequent clinical findings, including abdominal pain and CRP level (Fig. 4). Although oral prednisolone was initiated at 1 mg/kg/day in previous ECD case reports [5], we started oral prednisolone at 0.3 mg/kg/day (30 mg/day) in the outpatient setting because our patient did not show life-threatening manifestations and there was concern for adverse events such as infection due to high dose glucocorticoid. In fact, some improvement in renal function was observed after initiation of oral desmopressin for CDI (Fig. 4). While there is little data on the association between CDI and renal failure, it has been reported that treatment of diabetes insipidus with desmopressin can improve ischemic kidney injury [8]. In addition, diabetes insipidus can cause hydronephrosis and renal failure when treatment is delayed [9]. Thus, treatment of CDI by oral desmopressin may well have contributed to the overall improvement of renal function in this case. Interestingly, because the dose of desmopressin was not changed after initiation of prednisolone, glucocorticoid therapy may well be ineffective for pituitary lesions. Clinical manifestations of CDI in our patient persisted for ∼5 years before he came to our institution; it is therefore possible that long-term involvement of ECD in the pituitary lesions made CDI irreversible. It has been demonstrated that childhood Langerhans cell histiocytosis-induced CDI is irreversible unless the chemotherapy is initiated before CDI becomes fully established [10]. Further research on the efficacy of glucocorticoid is required to clarify organ-specific responses and optimal timing of treatment initiation.

In conclusion, we describe a case of ECD with BRAF V600E mutation and multiple organ involvement, including pituitary and retroperitoneal lesions, that was successfully treated by prednisolone. Glucocorticoid is therefore a plausible alternative for ECD with BRAF V600E mutation when the BRAF inhibitor vemurafenib cannot be used.

## Learning Points

- Erdheim-Chester disease (ECD) is a rare non-Langerhans cell histiocytosis characterized by xanthoma/xanthogranuloma infiltration in various organs and a broad spectrum of clinical presentations, including bone lesions, central diabetes insipidus (CDI), and renal failure. CDI is a relatively common endocrine disorder in ECD, occurring in 25% to 50% of cases.
- BRAF inhibitor therapy is recommended as first-line therapy for ECD patients with BRAF V600E mutation who also manifest cardiac/neurologic disease or end-organ dysfunction, but an alternative must be considered when vemurafenib cannot be used.
- Glucocorticoid can improve kidney dysfunction and FDG-PET findings and is a plausible alternative for ECD.

## Data Availability

Original data generated and analyzed during this study are included in this published article.

## Abbreviations

ADH: antidiuretic hormone
CDI: central diabetes insipidus
ECD: Erdheim-Chester disease
FDG: ^18^F-fluorodeoxyglucose
MAPK: mitogen-activated protein kinase
MRI: magnetic resonance imaging
PET: positron emission tomography
SUV max: maximum standardized uptake value
